# UF‐5000 flow cytometer: A new technology to support microbiologists’ interpretation of suspected urinary tract infections

**DOI:** 10.1002/mbo3.987

**Published:** 2020-01-06

**Authors:** Roberto Ippoliti, Isabella Allievi, Andrea Rocchetti

**Affiliations:** ^1^ Faculty of Business Administration and Economics University of Bielefeld Germany; ^2^ Microbiologia Azienda Ospedaliera SS. Antonio e Biagio e C. Arrigo Alessandria Italy

**Keywords:** flow cytometer, screening, sustainability of health treatments, urinary tract infection, urine culture

## Abstract

This case study aims to describe the adoption of an innovative flow cytometer (i.e., UF‐5000), which can support the microbiologists’ process of diagnosing suspected urinary tract infections (UTIs). The new clinical information provided can be used to improve the identification of both contamination and colonization, thus reducing inappropriate antibiotic prescriptions. In July and August 2017, the Microbiology Laboratory of Alessandria (Italy) conducted a retrospective monocentric study analyzing data about 1,295 urine specimens from inpatients and outpatients with symptoms of UTIs. The results of this study show that the innovative technology can successfully support the diagnostic process in microbiology laboratories and, consequently, the supply of sustainable treatments by hospitals.

## Abstract 2

This case study aims to describe the adoption of an innovative flow cytometer (i.e., UF‐5000), which can support the microbiologists’ process of diagnosing suspected urinary tract infections. The new clinical information provided can be used to improve the identification of both contamination and colonization, thus reducing inappropriate antibiotic prescriptions.

## 1 INTRODUCTION

Urinary tract infections (UTIs) are caused by pathogenic microorganisms, and they are recognized as among the most common infections in community and hospitalized patients (Stamm & Hooton, 1993; Monsen & Ryden, 2017). The standard tool used today to diagnose UTIs is urinary culture, despite the high prevalence of culture‐negative and false‐positive specimens (Broeren, Bahçeci, Vader, & Arents, 2011; de Frutos‐Serna et al., 2014; Pieretti et al., 2010). The low performance of the current diagnostic approach might be improved by adopting the flow cytometry technology (García‐Coca, Gadea, & Esteban, 2017). For example, Monsen and Ryden (2015) point out that the key information supplied by this technology can lead to a more appropriate evaluation of bacteriuria growth and count of white and red blood cells. Despite this evidence, the current literature focuses mainly on the adoption of flow cytometry analysis (FCA) in the screening of negative specimens to reduce urine cultures, while less attention is paid to the information that may be collected to support the interpretation of those cultures (Brilha, Proenca, Cristino, & Hänscheid, 2010; De Rosa et al., 2010; Okada et al., 2001). Considering the positive social and economic impact of appropriateness in antibiotic prescription, the medical community should consider incorporating the use of flow cytometry technology into the current standard interpretation process, supporting the microbiologists’ decision‐making.

The UF‐5000 is an automated urine analyzer produced by Sysmex Corporation, which performs FCA with a higher level of accuracy and more precise data (Seghezzi et al., 2017). This innovative analyzer can detect urine particles related to pathological processes of the urinary tract, providing the necessary clinical information to properly interpret specimens (Previtali, Ravasio, Seghezzi, Buoro, & Alessio, 2017). According to the current literature (Gieteling, Leur, Stegeman, & Groeneveld, 2014; Manoni et al., 2009; UK NHS, 2014), we regard the following as key clinical information necessary to distinguish between real UTI and contamination/colonization: squamous epithelial cells (SEC) and white blood cells (WBC). Urine conductivity is also analyzed in order to assess the quality of the specimens (De Rosa et al., 2010). Through this process, the UF‐5000 can improve the microbiologists’ interpretation activities, reducing the prevalence of false‐positive results in UTIs and supporting the sustainability of a key public service.

## 2 DATA AND METHODOLOGY

In July and August 2017, our team at the Microbiology Laboratory of the General Hospital of Alessandria (Italy) conducted a retrospective monocentric study analyzing data about 1,295 urine specimens from inpatients and outpatients with symptoms of UTIs. The need to investigate suspected UTIs (i.e., request to perform an examination), as reported by General Practitioners (GPs) or Hospital Practitioners (HPs), was the admission criterion for this study. No laboratory tests (e.g., dipstick test) were performed, and information about each patient's specific symptoms (e.g., whether low back pain and/or low abdominal pain were present, as well as frequency of the pain) was not available. In other words, our research team checked exclusively whether a GP or HP had submitted a request for diagnostic investigation due to suspected UTI, without further exploring the causes behind this request. Accordingly, all the other subjects were not included in the sample and not investigated. At the same time, the use of a catheter was an exclusion criterion, that is, all the samples considered were midstream urine specimens. As for the patients involved in this study, 55% were females, with an average age of 53, while the remaining 45% were males, with an average age of 60. Finally, 70% of the subjects were outpatients, while the other 30% were inpatients. Note that this is a retrospective analysis on anonymous data, previously collected by the hospital. Patients’ informed consents were routinely gathered before treatments and clinical procedures and, according to the national law, no formal ethical approval was needed.

Following Grosso, Bruschetta, Rosa, Avolio, and Camporese (2008), the specimens were collected in a sterile collection cup with an integrated device (Becton Dickinson) that allows the automated transfer of the urine to a vacutainer tube with preservatives (boric acid) and then to a vacutainer tube without preservatives for the automated urinalysis with UF‐5000. Significant bacteriuria was defined for *Escherichia coli* and *Staphylococcus saprophyticus* (but not for other pathogens) according to *Kass* criteria, and the samples were analyzed using Becton Dickinson CHROMagar Orientation (i.e., the standard laboratory procedure). The samples also underwent FCA using the UF‐5000 (i.e., the innovative laboratory procedure), to test whether this can provide greater accuracy, so as to support urine culture interpretation by microbiologists. Hence, the standard laboratory procedure represents the reference method adopted in this study, while the innovative laboratory procedure is a technique introduced to increase the available information that may be used to interpret the cultures correctly. The specimens were analyzed within 3 hr of arrival at the laboratory. The agar plates were incubated for 24 hr at 37°C in aerobic conditions and examined for significant bacteriuria. For what concerns the time span between collection of the urine specimens and delivery to the laboratory, we estimate it to be around 1 hr, for both inpatients and outpatients. Obviously, this is an estimation based on our experience, which might vary depending on specific personal circumstances for outpatients and/or the specific clinical cases for inpatients.

Table 1 shows some demographic characteristics of the total sample and the main uropathogens identified using the standard laboratory procedure (i.e., urine culture), in both absolute and percentage values. Table 2 proposes some descriptive statistics on the additional information collected using the innovative technology (i.e., UF‐5000): conductivity, SEC, and WBC.

**Table 1:** Demographic characteristics and urine culture data collected using the standard laboratory procedure

	*N* = 1,295	*n*	%
Culture results	Nonsignificant bacteriuria	988	76.3
	Contaminated	59	4.6
	Significant bacteriuria	248	19.2
Urine pathogens	*Escherichia coli*	150	60.5
	*Klebsiella pneumoniae*	22	8.9
	*Enterococcus faecalis*	19	7.7
	*Proteus mirabilis*	10	4.0
	*Pseudomonas aeruginosa*	8	3.2
	*Candida albicans*	5	2.0
	*Enterobacter cloacae*	5	2.0
	*Enterobacter aerogenes*	4	1.6
	Other microorganisms	25	10.1

**Table 2:** Additional information collected using the innovative laboratory procedure (i.e., flow cytometry technology)

Clinical information	Mean	*SD*	Min	Max
Squamous epithelial cells (SEC)	16.4	29.2	0.0	284.1
White blood cells (WBC)	466.5	2,073.5	0.0	29,273.0
Conductivity	13.6	6.2	0.0	34.9
*N* = 1,295				

In accordance with the European Confederation of Laboratory Medicine (ECLM)—European Urinalysis Group* (*Aspevall, Hallander, Gant, & Kouri, 2001), significant bacteriuria growth is defined as ≥10^3^ CFU/ml of primary pathogenic microorganisms, such as *E. coli* and *S. saprophyticus*. Based on our laboratory protocol, when growth is <10^3^ CFU/ml or, alternatively, mixed flora is detected in the cultures, the urine specimens are classified as contaminated (i.e., nonsignificant bacteriuria). Note that this laboratory, like many other microbiology laboratories, does not have the necessary data to evaluate borderline cases (e.g., 10^2^ CFU/mL > bacteriuria growth ≥ 10^3^ CFU/mL), in which only additional information (e.g., the patients’ clinical history) can support the microbiologists’ decision‐making process regarding suspected UTIs. Obviously, in this case, the microbiologists should involve the reference GPs or HPs in the diagnostic evaluation, as they can help interpret the results.

To achieve the proposed target, the team used the new flow cytometry technology to detect potential contamination and/or colonization in the specimens, which can support microbiologists in the interpretation of bacteriuria growth. Indeed, the literature indicates that bacteriuria growth is not sufficient to identify UTIs and that the count of white blood cells and epithelial cells is necessary for appropriate diagnosis, as well as to identify potential contamination (Liou, Currie, James, Malone‐Lee, & David, 2017; Monsen & Ryden, 2015). Accordingly, even when significant bacteriuria growth was detected (≥10^3^ CFU/mL), the specimens were classified as negative if SEC >30/µl or WBC <5/µl. Even more importantly, urine conductivity is essential in determining whether a specimen was collected during the prescribed time period, thus ensuring the expected quality level. According to the current literature (De Rosa et al., 2010), conductivity represents an instrumental parameter allowing the indirect evaluation of electrolytic concentration in urine, measured by its electrical conduction capacity and expressed in mS/cm. It is measured before the cells pass through the flow cell and it is an expression of renal diuresis, that is, the ability of the kidneys to concentrate urine. Hence, conductivity can be regarded as a good parameter to check the quality of sampling—and it is measured by the new technology under investigation (i.e., UF 5000). Its acceptability threshold is set at 6 mS/cm, that is to say, a sample with a value <6 mS/cm is considered diluted, leading to no further clinical investigation and, consequently, to its exclusion.

Therefore, although significant bacteriuria growth was detected (≥10^3^ CFU/ml), the specimens were classified as negative if SEC >30/µl, or urine conductivity <6 mS/cm, or WBC <5/µl. These are the thresholds adopted by the microbiologists involved in our case study to support their decision‐making process in diagnosing UTIs.

## 3 RESULTS

Our results show that 19.15% of the urine specimens analyzed by the team presented significant bacteriuria growth. However, 27.82% of these specimens did not satisfy the necessary conditions to exclude potential contamination and/or colonization, according to the selected key information mentioned above. Specifically, the team looked at all the specimens with bacteriuria growth ≥10^3^ CFU/ml and found that 20 of them had urine conductivity <6 mS/cm, while 34 specimens had SEC >30/µl, and 17 specimens had WBC <5/µl. Based on the proposed thresholds, these specimens were considered negative and, therefore, discarded.

Using the additional information made available through the innovative procedure (i.e., conductivity, SEC, and WBC), we estimated the diagnostic effectiveness of our standard laboratory procedure (i.e., urine culture), which clearly represents the current gold standard. In detail, considering primary pathogenic microorganisms, such as *E. coli* and *S. saprophyticus*, we calculated both specificity (SP) and sensitivity (SE) in identifying an UTI based on the available information (i.e., significant bacteriuria). These data were compared with the microbiologists’ interpretation of bacteriuria growth based on the additional information supplied by the UF‐5000 flow cytometer and the suggested thresholds for negative specimens (i.e., SEC > 30/µl, or urine conductivity < 6 mS/cm, or WBC < 5/µl). Table 3 displays our results.

**Table 3:** Diagnostic accuracy performance of the current gold standard (i.e., urine culture)

*N* = 1,295	
Sensitivity (SE)	1.0
Specificity (SP)	0.9
Positive likelihood ratio (LR+)	16.2
Negative likelihood ratio (LR−)	0.0
Accuracy	0.9
Area under the curve (AUC)	1.0
Positive predictive value (PPV)	0.7
Negative predictive value (NPV)	1.0

According to our results, sensitivity is equal to 100% and specificity is equal to 94%, with a total of 69 false positives. Finally, the area under the curve (AUC) is 0.9691, while the positive predictive value (PPV) is equal to 72% and the negative predictive value (NPV) is equal to 100%.

### 3.1 Limits

Although our results are quite interesting, there are some limits to the analysis proposed here. Indeed, no information was available to us about the symptoms related to these suspected UTIs, which could be key in interpreting the results. The reader might argue that, considering the presence of symptoms (admission criterion), the percentage of specimens with significant bacteriuria growth is quiet low (19.15%). A possible explanation might have to do with heterogeneity in how GPs and HPs interpret the symptoms reported. This could be avoided by performing laboratory tests (e.g., dipstick test) prior to inclusion in the study. Nevertheless, the absence of available funds and human resources forced the research team to perform these activities.

## 4 CONCLUSIONS

The current age of austerity and the related spending review policies affect national and local budgets, driving public healthcare systems to use the scarce resources available even more rationally (Ippoliti et al., 2018; Quaglio, Karapiperis, Woensel, Arnold, & McDaid, 2013; Stuckler, Reeves, Loopstra, Karanikolos, & McKee, 2017). The results of this research suggest that the microbiology community should consider the advantages offered by the flow cytometry technology and its additional information on urinary specimens. This new technology, specifically the UF‐5000, can successfully support healthcare professionals in the diagnostic process by identifying infections in the urine samples of symptomatic patients with high levels of accuracy. If this new technology is not adopted by microbiology laboratories, existing patients may be exposed to false‐positive results, which lead to the unnecessary use of antibiotics.

## CONFLICT OF INTEREST

None declared.

## AUTHOR CONTRIBUTIONS

Roberto Ippoliti; Formal analysis‐Lead, Methodology‐Lead, Writing‐original draft‐Lead, Writing‐review & editing‐Lead. Isabella Allievi; Data curation‐Supporting, Writing‐original draft‐Supporting, Writing‐review & editing‐Supporting. Andrea Rocchetti; Conceptualization‐Lead, Data curation‐Lead, Investigation‐Lead, Writing‐original draft‐Supporting, Writing‐review & editing‐Supporting.

## ETHICS STATEMENT

None required.

## Data Availability

The datasets used and/or analyzed during the current study are available from the corresponding author on reasonable request.
